# Closing the adaptive window: immune–metabolic strategies to constrain melanoma therapy resistance

**DOI:** 10.3389/fimmu.2026.1954638

**Published:** 2026-10-20

**Authors:** Federica Riccardo, Laura Conti, Paola Defilippi, Pietro Arina, Valentina Audrito, Enzo Calautti

**Affiliations:** 1 Department of Molecular Biotechnology and Health Sciences, Molecular Biotechnology Center “Guido Tarone”, University of Turin, Torino, Italy; 2 Bloomsbury Institute of Intensive Care Medicine and Human Physiology and Performance Laboratory, Centre for Perioperative Medicine, Department of Targeted Intervention, University College London, London, United Kingdom; 3 Department of Science and Technological Innovation, University of Eastern Piedmont, Alessandria, Italy

**Keywords:** adaptation, melanoma, metabolism, precision medicine, signaling, therapy resistance, tumor microenvironment

## Abstract

Melanoma cells respond to therapy not only through selection of pre-existing resistant clones, but also by rapidly engaging reversible adaptive programs that preserve viability under treatment. These programs connect signaling rewiring, metabolic plasticity, inflammatory responses, cell-state transitions and remodeling of the tumor microenvironment. We propose that this early adaptive phase creates a temporal and biological window in which melanoma cells acquire sufficient fitness to persist and subsequently evolve more stable epigenetic and genetic resistance. We first examine the rapid signaling responses elicited by MAPK pathway inhibition and the adaptive programs induced by immune-mediated pressure, emphasizing multifunctional hubs that integrate growth, stress, metabolic and inflammatory cues. We then discuss the metabolic flexibility of melanoma cells, including mitochondrial respiration, glutamine and lipid utilization, redox control and NAD/NAMPT-dependent immunometabolic regulation. Finally, we consider therapeutic vaccination and microbiome modulation as complementary strategies that may broaden anti-tumor immunity and reshape treatment responsiveness. Thus, this review focuses primarily on convergent adaptive processes that define this biological window and explores whether their experimental characterization may reveal opportunities to therapeutically constrain adaptive capacity through rationally timed combinations or sequential interventions. Closing the adaptive window may reduce the evolutionary opportunities available to melanoma cells before heterogeneous, fixed resistance mechanisms become established.

## 1 Introduction

Melanoma has become one of the most successful examples of precision oncology. Targeted therapies directed against the MAPK pathway and immune checkpoint inhibitors (ICIs) have profoundly improved the management of advanced disease, producing unprecedented gains in progression-free and overall survival (1). Nevertheless, primary and acquired resistance remain the principal obstacles to durable clinical benefit, and understanding how melanoma escapes therapeutic control remains a major challenge.

The biological basis of therapeutic resistance has been extensively investigated, revealing genetic, epigenetic, metabolic and microenvironmental mechanisms that promote tumor persistence. Several comprehensive reviews have summarized these mechanisms and their clinical implications (2–4). Building upon these foundations, the present Review focuses on early adaptive processes engaged following therapeutic perturbation that coordinate signaling, metabolism, inflammatory responses and cell-state plasticity (5, 6). We consider these processes as interconnected programs that shape melanoma evolution under treatment.

Therapeutic agents are commonly regarded as cytotoxic or cytostatic interventions whose primary function is to eliminate tumor cells. However, they also disrupt cellular homeostasis and trigger coordinated biological responses (7). Therapy-sensitive cells fail to restore fitness and undergo irreversible arrest or death. Surviving cells instead remodel signaling, metabolism, transcription and interactions with the tumor microenvironment (TME), thereby maintaining sufficient viability under continued therapeutic pressure.

Accumulating evidence indicates that such adaptive responses frequently precede genetically encoded resistance (8). Mutations, gene amplifications and other stable alterations ultimately drive many clinically resistant melanomas, but tumor cells must first survive the acute stress imposed by treatment. Adaptive remodeling of signaling networks, metabolism, chromatin and lineage identity may therefore create a permissive state in which heterogeneous resistance mechanisms emerge and undergo positive selection.

The adaptive window hypothesis is not intended to define a new cellular state or a resistance mechanism distinct from drug-tolerant persistence, reversible adaptive resistance or phenotypic plasticity. Rather, these processes can be viewed as overlapping manifestations of adaptation to therapeutic stress: in this context, drug-tolerant persistence refers to the survival of tumor-cell populations under therapeutic pressure without stable resistance, phenotypic plasticity to the capacity of tumor cells to transition between functional states, and reversible adaptive resistance to therapy-induced changes that transiently reduce drug sensitivity without becoming stabilized into durable resistance. We conceptualize the adaptive window as a biologically defined and temporally dynamic phase during which tumors retain the capacity to access alternative viable adaptive trajectories under therapeutic pressure. This view is consistent with single-cell and perturbational studies showing co-existing drug-tolerant states, distinct trajectories toward drug tolerance, and experimentally modifiable priming and cell-state transitions (9–12). Importantly, the adaptive window hypothesis generates a testable prediction beyond the identification of individual persister states or phenotypic transitions: experimental perturbations that restrict adaptive capacity should reduce the diversity or accessibility of viable adaptive trajectories, rather than merely suppressing the adaptive response that is dominant at a given time.

This framework may have direct therapeutic implications. Whereas fixed resistance mechanisms vary substantially among patients and even among lesions from the same patient, early adaptive responses may converge on a more restricted set of biological processes and dependencies. Therapeutic strategies could therefore aim not only to inhibit an established escape mechanism, but to constrain the adaptive possibilities available to the tumor, thereby limiting the stabilization of adaptive responses into durable resistance. This may require targeting shared enabling dependencies or complementary adaptive routes prior to resistance stabilization, using combinations or sequential interventions timed to the adaptive changes induced by the initial therapy.

The efficacy of a given therapeutic combination may depend on when the intervention is introduced relative to the evolving adaptive state. An intervention administered before a relevant adaptive program is engaged, during its active phase, or after resistance has become stabilized may therefore produce different biological and therapeutic effects. The framework specifically predicts that targeting an adaptive dependency while it remains reversible may restrict subsequent trajectories more effectively than targeting the same dependency after stable resistance has emerged. This provides a direct experimental comparison between fixed-timing, adaptation-guided and post-resistance intervention (8, 13, 14).

Accordingly, we examine adaptive signaling networks and the metabolic and inflammatory programs that sustain drug-tolerant states. We also discuss complementary therapeutic approaches—including metabolic intervention, immunotherapy, vaccination and microbiome modulation—that may interfere with these trajectories (**Figure 1**). The central therapeutic challenge is to prevent melanoma cells from rebuilding a viable state after treatment-induced perturbation.

**Figure 1:**
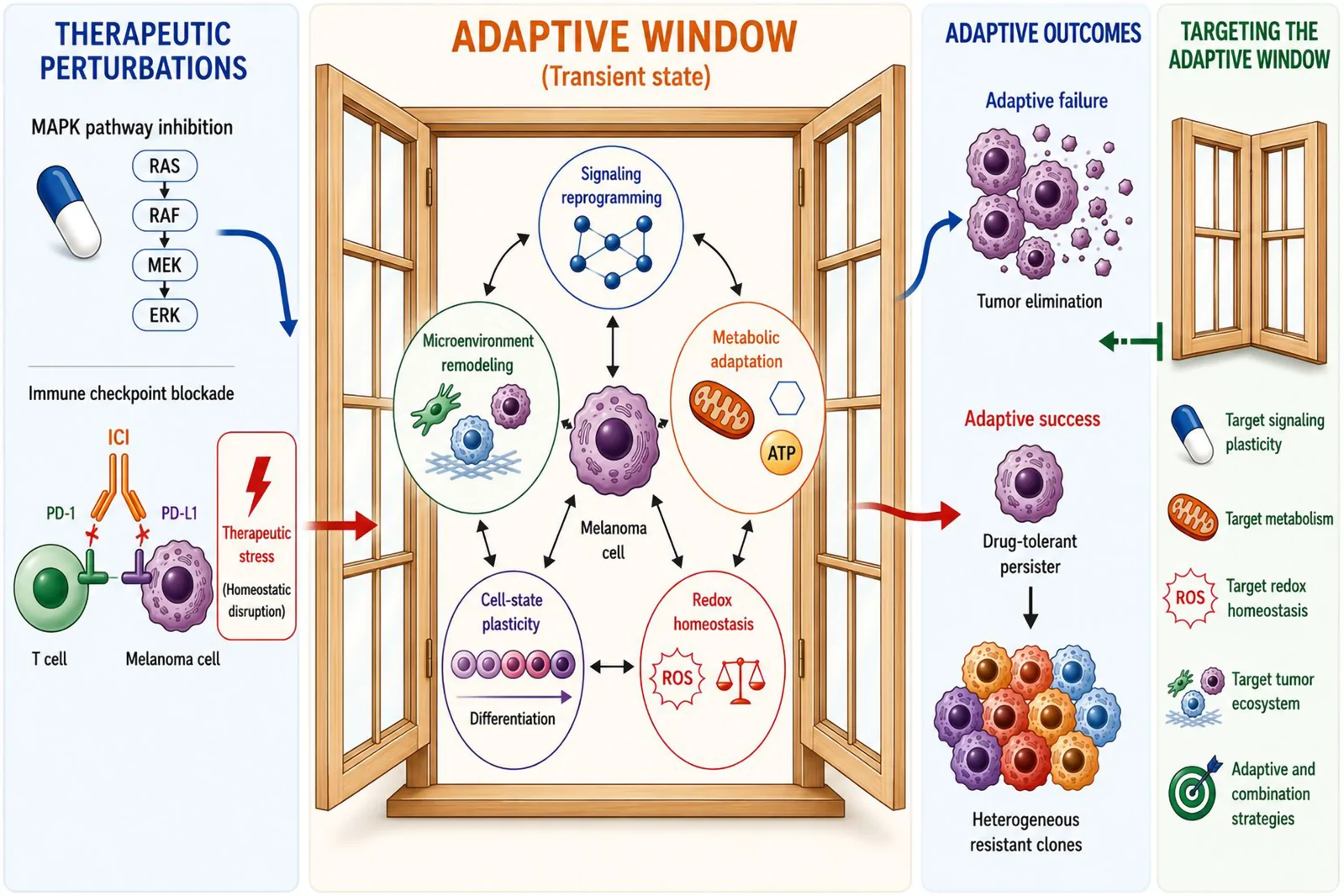
Graphical summary of the adaptive window concept in melanoma therapy resistance. This figure serves as the graphical summary of the review, illustrating the conceptual framework that underpins the manuscript. Therapeutic perturbations, exemplified by MAPK pathway inhibition and immune checkpoint blockade, induce therapeutic stress and transiently open an adaptive window, a dynamic adaptive phase, in which melanoma cells undergo coordinated signaling, metabolic, redox, microenvironmental, and cell-state reprogramming. Depending on the success or failure of this adaptive process, tumor cells either undergo elimination or survive as drug-tolerant persisters that may ultimately evolve into heterogeneous resistant clones. The progressive closure of the adaptive window symbolizes therapeutic strategies aimed at constraining cellular plasticity before stable resistance is established.

## 2 Adaptive signaling networks orchestrating melanoma adaptation

The two principal therapeutic arms used in advanced melanoma—MAPK-targeted therapy and ICIs—generate different primary pressures but rapidly engage overlapping adaptive biology. MAPK inhibitors suppress an oncogenic dependency, whereas ICIs intensify immune-mediated and inflammatory pressure. *In vivo*, however, neither perturbation occurs in isolation: melanoma cells integrate changes in intracellular signaling, metabolism, cytokine exposure, immune surveillance, extracellular matrix composition and nutrient availability, as described in this and in the following metabolic section and summarized in **Figure 2**. This section follows the temporal and functional logic of adaptation, emphasizing signaling modules that coordinate multiple biological outputs (15).

**Figure 2:**
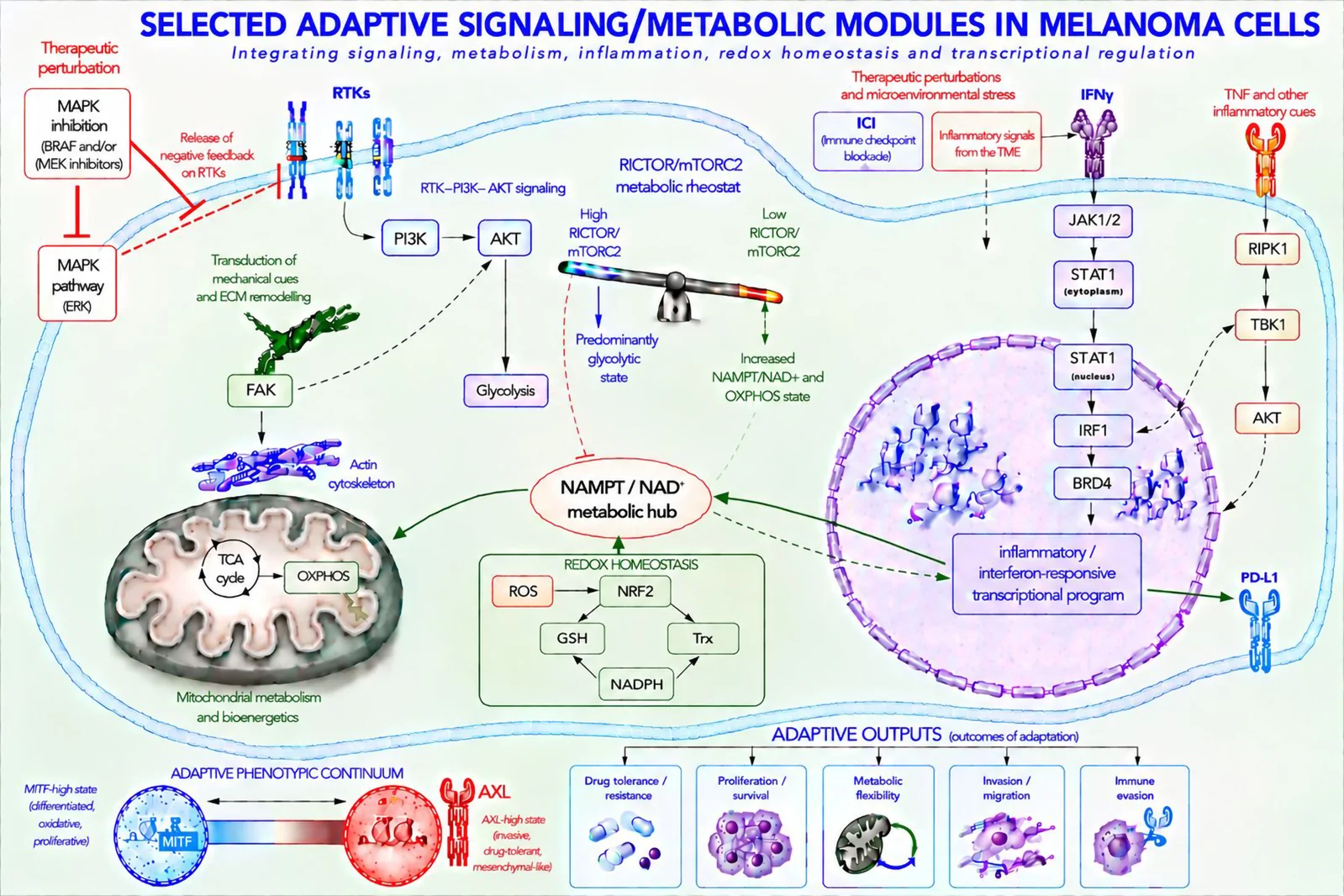
Selected adaptive signaling/metabolic modules in melanoma cells. Simplified framework illustrating representative signaling, metabolic, inflammatory and cell-state programs engaged during melanoma adaptation to therapeutic and microenvironmental pressures. The RICTOR/mTORC2 rheostat summarizes the functional association between RICTOR/mTORC2 activity, glycolytic metabolism and NAMPT/NAD^+^-dependent mitochondrial metabolism; the intermediate mechanisms remain incompletely defined. The NAMPT/NAD^+^ hub and MITF-high ↔ AXL-high continuum illustrate metabolic integration and reversible phenotypic plasticity, respectively. Solid connecting lines indicate established molecular and functional relationships, whereas dashed connecting lines indicate experimentally supported functional relationships whose underlying molecular mechanisms remain incompletely defined. Arrowheads and blunt-ended connectors indicate activating and inhibitory relationships, respectively.

### 2.1 Early adaptation to MAPK pathway inhibition: from oncogene addiction to adaptive rewiring

BRAFV600E-mutant melanoma is a paradigmatic example of oncogene addiction (16, 17), in which constitutive MAPK signaling becomes a dominant regulator of proliferation, anabolic metabolism, lineage maintenance and survival. The efficacy of BRAF and MEK inhibitors reflects this dependence; conversely, abrupt ERK suppression creates an immediate homeostatic crisis. Some ensuing events are direct and largely unavoidable consequences of target inhibition, whereas the compensatory routes used by surviving cells depend on their pre-existing transcriptional and epigenetic state, genotype and microenvironment.

Effective BRAF/MEK inhibition rapidly suppresses ERK-dependent transcription, resulting in a profound reduction in anabolic metabolism, protein synthesis and proliferative output (18, 19). Paired patient biopsies showed that early suppression of mTORC1 output, monitored by decreased ribosomal protein S6 phosphorylation, correlates with clinical response, indicating that metabolic and biosynthetic collapse is an early pharmacodynamic consequence of MAPK inhibition (19). At the same time, ERK inhibition releases negative-feedback mechanisms that normally restrain upstream signaling, restoring responsiveness to growth factors and creating permissive conditions for alternative survival pathways (20).

One of the earliest and most recurrent adaptive responses is reactivation of PI3K–AKT signaling through receptor tyrosine kinases (RTKs). Early on-treatment biopsies from patients receiving BRAFi or combined BRAFi/MEKi frequently showed rebound AKT phosphorylation despite sustained MAPK suppression, establishing AKT activation as an early adaptive response rather than solely a feature of fully resistant tumors (21). PDGFRβ, EGFR, ERBB3, IGF1R, MET, AXL and other receptors, with emerging evidence implicating new players such as the chondroitin sulfate proteoglycan 4 (CSPG4), can contribute to this response (22–26). Although the RTKs involved vary across models and patients, their activation frequently converges on restoration of PI3K–AKT-dependent survival signaling.

Adaptive rewiring also recruits adhesion- and mechanotransduction-dependent pathways. A recent study showed that MAPK inhibition reduces expression of the ERK-regulated RhoA antagonist RND3/RhoE, thereby activating the RhoA–ROCK–FAK–AKT axis. FAK inhibition enhanced responses to MAPK-targeted therapy and overcame resistance in melanoma models derived from patients progressing on targeted or immune therapies, providing a mechanistically defined and druggable example of convergence between these therapeutic contexts (14).

mTORC2/RICTOR provides a distinct example of adaptive signaling rewiring during MAPK-targeted therapy. Although mTORC2 is generally regarded as a positive regulator of AKT signaling and cell survival (27), its contribution to therapeutic adaptation is highly context dependent. RICTOR undergoes proteasome-dependent downregulation following vemurafenib exposure, identifying mTORC2 as a dynamic target of the adaptive response rather than a static signaling component. Although reduced RICTOR expression has limited consequences under basal growth conditions, it enhances the survival and proliferative capacity of BRAF-mutant melanoma cells exposed to MAPK inhibition by promoting adaptive rewiring toward an oxidative phosphorylation- and nicotinamide phosphoribosyltransferase (NAMPT)-dependent state (28). These findings illustrate that therapy-induced remodeling of a signaling node does not necessarily abolish critical downstream functions but may instead redistribute cellular dependencies and generate context-specific vulnerabilities. The metabolic consequences of this rewiring and their therapeutic implications are discussed further in Section 3.

The initial signaling response is subsequently incorporated into broader stress-adaptive programs that reshape cellular physiology and identity. Stress-responsive transcription factors, including the JNK–c-JUN axis, reduce apoptotic sensitivity and contribute to phenotypic plasticity during both early and established drug-tolerant states (29–31). In parallel, energetic stress activates sensors such as 5’ AMP-activated protein kinase (AMPK), coupling diminished anabolic activity to autophagy and other survival programs under MAPK pathway inhibition (32). Activation of the integrated stress response, translational remodeling and widespread chromatin reprogramming further generate heterogeneous transcriptional states from which drug-tolerant persister populations can emerge (33–35). Together, these processes reshape melanoma cell identity and establish the biological framework from which more stable drug-tolerant states may develop.

Thus, the earliest adaptive phase following MAPK pathway inhibition is characterized by common constraints—loss of ERK output, feedback release, reduced anabolic support and energetic stress—followed by heterogeneous patterns of signaling rewiring. Despite engaging different upstream receptors and mechanotransduction pathways, these responses converge on preservation of sufficient survival signaling to maintain viability during the initial therapeutic perturbation. Durable adaptation, however, requires the subsequent coordination of stress responses, inflammatory cues, metabolic remodeling and cell-state plasticity.

### 2.2 Adaptation to immune-mediated inflammatory pressure

Immune-mediated inflammatory pressure represents a fundamentally distinct form of therapeutic stress. Unlike MAPK-targeted therapy, which directly perturbs melanoma-intrinsic oncogenic signaling, immune checkpoint inhibition interferes with adaptive immune resistance mechanisms that have already developed within the TME.

PD-1/PD-L1 signaling constitutes a physiological negative-feedback circuit induced by inflammatory cytokines, particularly interferon (IFN)-γ, which limits excessive immune activation while simultaneously protecting tumor cells from immune-mediated killing (36). Anti-PD-1 therapy therefore targets an adaptive immune resistance program in addition to reinvigorating anti-tumor T-cell activity.

Following checkpoint blockade, melanoma cells are exposed to an altered inflammatory milieu characterized by increased IFN-γ, Tumor Necrosis Factor (TNF) and other immune-derived mediators. Although mechanistically distinct from MAPK inhibition, this environment imposes related biological constraints. Tumor cells must preserve viability despite sustained inflammatory stress, maintain metabolic homeostasis, modulate lineage identity and avoid irreversible activation of cell-death programs.

Among these inflammatory signals, IFN-γ exemplifies the dual nature of therapeutic adaptation. Acute IFN-γ signaling enhances antigen processing and presentation, promotes the recruitment and activation of effector lymphocytes and can directly inhibit melanoma proliferation. Persistent IFN-γ exposure, however, can induce adaptive resistance programs including PD-L1 expression, remodeling of JAK–STAT signaling, impaired antigen presentation and selection of dedifferentiated, inflammation-tolerant cellular states (5, 36, 37). The biological consequences of IFN-γ therefore depend not only on signal intensity and duration, but also on lineage state, genetic context and the adaptive capacity of the responding cell.

TNF represents a complementary inflammatory challenge. Depending on cellular context, TNF signaling can promote tumor elimination or support adaptation and survival. Receptor-interacting serine/threonine-protein kinase 1 (RIPK1) has been identified as an IFN-γ-inducible regulator contributing to inflammatory adaptation and resistance to immune checkpoint blockade, illustrating how sustained interferon signaling can modify the subsequent interpretation of TNF-family receptor signals (38).

Inflammatory adaptation is not unique to immune checkpoint therapy. MAPK inhibition can transiently enhance melanoma antigen expression, T-cell infiltration and immune recognition, whereas prolonged exposure may promote dedifferentiation, antigen loss and inflammatory programs associated with immune escape (5, 37, 39). Thus, although targeted therapy and immune checkpoint blockade impose distinct initiating pressures, they may ultimately generate adaptive states characterized by increasing coordination of inflammatory, metabolic and stress-response programs (12, 40).

### 2.3 Multifunctional signaling nodes coordinating adaptive responses

This convergence raises a broader question: how are diverse biological cues coordinated within melanoma cells? Therapeutic adaptation emerges through the functional integration of mechanical, inflammatory, metabolic and stress-associated inputs into coherent cellular responses. Focal adhesion kinase (FAK), TANK-binding kinase 1 (TBK1) and RIPK1 illustrate complementary modes of such coordination across tumor–microenvironment communication, intracellular stress and inflammatory signaling, and cell-fate control. Their multifunctionality allows adaptive responses to extend beyond the pathways in which these proteins were initially characterized.

Tumor–microenvironment communication provides a first mode of adaptive coordination. Functioning both as a kinase and as a multidomain signaling scaffold, FAK occupies a strategic position at the interface between extracellular architecture and intracellular signaling. It integrates adhesive, mechanical and growth factor-derived signals to regulate cell survival, cytoskeletal remodeling, migration and invasion (41). Beyond these tumor-intrinsic functions, FAK shapes multiple components of the TME by regulating extracellular matrix remodeling, stromal organization and the recruitment or maintenance of immunosuppressive myeloid and regulatory T-cell populations (42). FAK inhibition can therefore impair tumor-cell survival while also remodeling the TME toward a state more permissive for anti-tumor immunity and enhancing responses to immune checkpoint blockade in preclinical models (42, 43).

As discussed in Section 2.1, MAPK inhibition engages this multifunctional architecture through activation of the RhoA–ROCK–FAK axis, illustrating how therapy-induced rewiring can co-opt signaling systems that normally integrate extracellular and intracellular cues. Accordingly, FAK inhibition is being explored as a strategy to limit adaptive resistance, particularly in combination with MAPK-targeted therapies or immune checkpoint blockade in melanoma (14, 41, 43).

TBK1 provides a broader example of adaptive signal coordination. Although initially characterized as a central regulator of innate immune responses and type I IFN signaling, TBK1 is recruited or activated in response to signals arising from nucleic-acid sensing, inflammatory and oncogenic pathways, as well as mitochondrial stress, hypoxia and autophagic processes (44–46). Its downstream functions extend well beyond interferon production and include regulation of NF-κB signaling, selective autophagy and mitochondrial quality control, mTORC1 activity and cellular metabolism. TBK1 can also directly promote AKT phosphorylation and activation independently of PDK1 and mTORC2, providing an additional connection between innate immune and pro-survival signaling (47).

TBK1 activation may therefore reflect broad adaptive network remodeling extending beyond type I interferon signaling alone. A recent study identified TBK1 as a tumor-intrinsic determinant of resistance to immune checkpoint blockade. Across experimental and patient-derived melanoma models, TBK1 inhibition enhanced responses to PD-1 blockade by lowering the cytotoxicity threshold to TNF and IFN-γ, thereby sensitizing tumor cells to inflammatory cell death (48).

The IFN-γ-inducible role of RIPK1 introduced above provides a complementary example of signal coordination at the level of cell-fate control. RIPK1 links interferon-driven transcriptional adaptation to the downstream interpretation of TNF-family receptor inputs. Through its recruitment to distinct signaling complexes and its separable scaffolding and kinase functions, RIPK1 can direct inflammatory signals toward NF-κB- and MAPK-dependent survival or toward apoptosis and necroptosis (49, 50).

In experimental melanoma models, tumor-cell RIPK1 favored pro-survival TNF signaling and an immunosuppressive chemokine program, thereby promoting both cell-intrinsic resistance to immune-mediated killing and cell-extrinsic remodeling of the TME. Conversely, RIPK1 deletion favored CASP8-dependent tumor-cell killing, reduced arginase 1 (ARG1^+^) suppressive myeloid populations, increased T- and NK-cell infiltration and improved responses to immune checkpoint blockade. Notably, these resistance functions depended on RIPK1 scaffolding rather than its kinase activity (38).

This cell-fate checkpoint is also regulated by TBK1 and canonical IKK signaling, which restrain RIPK1-dependent cytotoxicity and contribute to the threshold at which inflammatory signaling becomes lethal (48). RIPK1 therefore lies at the intersection of interferon-driven adaptation, TNF-dependent survival and inflammatory cell death, illustrating how sustained immune pressure can be converted into both tumor-cell survival and microenvironmental immunosuppression.

Collectively, these nodes illustrate a broader principle: adaptation emerges through the reorganization of interconnected signaling networks rather than through the activation of isolated pathways. Multifunctional signaling nodes may offer opportunities to disrupt several adaptive outputs simultaneously. The same pleiotropy, however, means that their inhibition may produce divergent effects across tumor, immune and normal tissue compartments. Their therapeutic value may therefore depend as much on timing and sequence as on target selection: early intervention could prevent the establishment of adaptive tolerance, whereas sequential treatment may exploit vulnerabilities generated by the initiating therapy.

### 2.4 From reversible adaptation to stabilized resistant states

Early signaling and metabolic responses are dynamic and frequently reversible (51). Drug withdrawal can restore sensitivity in selected models, and single-cell studies demonstrate movement among transcriptional states rather than progression along a single irreversible trajectory (12). Persistent therapeutic pressure, however, can stabilize adaptive phenotypes through altered transcription-factor networks, chromatin remodeling and selection of genetic changes.

Phenotypic plasticity feeds back on the adaptive network by changing receptor expression, metabolism, antigen presentation and inflammatory sensitivity. Microphthalmia-associated transcription factor (MITF)-high oxidative cells, MITF-low/AXL-high invasive populations, neural crest-like states and slow-cycling persisters possess distinct functional dependencies (8, 52, 53). Epigenetic remodeling can provide cellular memory of initially transient signals (35). Because chromatin-modifying enzymes depend on metabolites such as acetyl-CoA, α-ketoglutarate and NAD, metabolic adaptation may also participate directly in consolidating drug tolerance (54).

These non-genetic states provide both time and a permissive context for heterogeneous genetic alterations that reactivate MAPK signaling or engage PI3K–AKT and other survival pathways. The early adaptive phase may therefore be substantially more convergent than the stable genetic endpoints that ultimately emerge.

Within the adaptive window hypothesis, these observations distinguish the adaptive state that is observed at a given time from the broader capacity of the tumor to access viable adaptive states. Drug-tolerant persister cells and phenotypic switching are therefore not competing definitions of the window, but experimentally observable manifestations of adaptation whose prevalence, accessibility and stability can change during treatment.

## 3 Metabolic plasticity and immunometabolic crosstalk

Metabolic reprogramming is a central component of melanoma adaptation rather than a fixed hallmark of resistant cells. Melanoma populations can redistribute carbon sources, mitochondrial activity and redox defenses in response to nutrient availability, hypoxia, therapy and immune pressure. This flexibility sustains drug-tolerant states and simultaneously modifies the TME, linking tumor bioenergetics to immune function, see **Figure 2** (55–59).

### 3.1 From glycolytic dependence to mitochondrial flexibility

Constitutive MAPK signaling promotes glucose uptake, glycolysis and anabolic metabolism in BRAF-mutant melanoma (57, 60, 61). BRAF/MEK inhibition initially suppresses glycolytic flux, contributing to energetic stress and apoptosis in sensitive cells. Surviving populations can progressively increase mitochondrial mass, respiratory capacity and tricarboxylic acid (TCA)-cycle activity, frequently through reactivation of the MITF–proliferator-activated receptor γ coactivator 1 alpha (PGC-1α) program (62, 63). Oxidative phosphorylation (OXPHOS) supports ATP production and redox control when glycolysis is curtailed and is among the best-characterized adaptations to MAPK inhibition (64, 65).

OXPHOS is nevertheless not a universal endpoint. Some persister populations enter a low-glycolysis/low-respiration state that limits biosynthetic demand, whereas others rely on active mitochondrial respiration, fatty-acid oxidation or alternative extracellular metabolites. The conserved feature is therefore metabolic flexibility rather than use of one obligatory pathway (56, 58, 66).

### 3.2 Glutamine, lipids and redox buffering

Mitochondrial adaptation requires continuous anaplerosis. Resistant melanoma cells often increase glutamine utilization to replenish TCA-cycle intermediates, maintain glutathione and generate NADPH (67, 68). Glutaminase inhibition can reduce respiration and viability in resistant models (67), although pyruvate carboxylase and other anaplerotic routes may compensate, illustrating why inhibition of a single nutrient pathway may be insufficient.

Lipid metabolism provides another source of acetyl-CoA and reducing equivalents (69, 70). Slow-cycling and drug-tolerant populations may increase fatty-acid uptake or oxidation, including ACOX1-dependent programs (71). Acquired vemurafenib resistance has also been linked to inflammatory lipid metabolism and prostaglandin production, connecting metabolic rewiring to tumor-promoting inflammation (69).

Increased mitochondrial use generates reactive oxygen species (ROS), making redox control indispensable. Resistant and persister cells activate glutathione, thioredoxin, NADPH-generating and Nuclear Factor Erythroid 2-related factor 2 (NRF2)-dependent defenses. This creates a potential vulnerability: overwhelming oxidant capacity or inhibiting NRF2-associated programs can restore sensitivity in selected models (64, 72).

### 3.3 NAD/NAMPT as a metabolic determinant of adaptive persistence

NAD availability connects mitochondrial respiration, redox control, DNA repair and chromatin regulation. NAMPT, the rate-limiting enzyme of the NAD salvage pathway, is frequently upregulated in melanoma and supports resistance to MAPK-targeted therapy. Pharmacological or genetic inhibition of NAMPT reduces melanoma viability and can enhance MAPK inhibition, whereas NAMPT overexpression recapitulates features of the resistant phenotype (73, 74). NAMPT and nicotinamide N-methyl transferase (NNMT) may also act extracellularly, either as soluble proteins or within extracellular vesicles, thereby remodeling stromal and immune compartments (75).

The adaptive mechanisms leading to increased NAMPT activity are likely to differ among melanoma lineages, suggesting that distinct molecular routes converge on a common requirement for sustained NAD salvage. One illustrative example is provided by therapy-induced downregulation of the mTORC2 component RICTOR. As discussed in Section 2.1, RICTOR protein undergoes proteasome-dependent downregulation following BRAF inhibition. Beyond its canonical role in AKT signaling, mTORC2 is a major regulator of glucose utilization and anabolic metabolism (27). Under the profound glycolytic suppression imposed by MAPK inhibition, reduced RICTOR expression may therefore facilitate the transition toward mitochondrial oxidative metabolism, increasing cellular dependence on continuous NAD regeneration. Consistent with this model, RICTOR-deficient melanoma cells display enhanced mitochondrial respiration, increased NAMPT dependence and greater resistance to BRAF/MEK inhibition. Moreover, transcriptomic analysis of TCGA cutaneous melanoma cohorts revealed coordinated enrichment of genes involved in mitochondrial ATP production in tumors expressing low levels of RICTOR, suggesting that this metabolic configuration extends beyond experimental models (28).

Together, these observations support the concept that therapy-induced signaling rewiring and metabolic adaptation converge on NAD metabolism as a critical determinant of melanoma persistence under therapeutic stress.

### 3.4 Drug-tolerant persister cells as a heterogeneous mix of metabolic states

Drug-tolerant persister cells are reversible, non-genetically resistant populations that survive prolonged therapy and can seed relapse. They should not be viewed simply as dormant residual cells: persisters occupy heterogeneous metabolic states (76–78). Some suppress both glycolysis and OXPHOS, whereas others retain mitochondrial respiration, fatty-acid oxidation or enhanced antioxidant defenses (66). Their defining property is the capacity to adjust energy expenditure and substrate use while avoiding lethal stress.

This heterogeneity limits the value of a single universal metabolic target. A more effective strategy may combine primary therapy with interventions selected against the dependencies induced within particular persister states. Alternatively, it may target shared requirements such as redox buffering and NAD homeostasis before epigenetic and genetic resistance becomes fixed.

### 3.5 Immunometabolic crosstalk within the TME

Therapeutic resistance is not determined solely by tumor-intrinsic metabolic adaptations but emerges from the continuous metabolic interplay between melanoma cells and the diverse cellular components of the TME (79–84), summarized in **Figure 3**. Tumor metabolism and immune regulation are tightly interconnected, forming an immunometabolic network in which malignant cells reshape nutrient availability, oxygen tension, extracellular pH, and redox balance, while stromal and immune cells reciprocally influence tumor evolution and therapeutic response. Consequently, metabolic rewiring extends beyond the cancer cell itself and progressively remodels the entire cellular ecosystem of the TME (59, 80, 83, 85–92).

**Figure 3:**
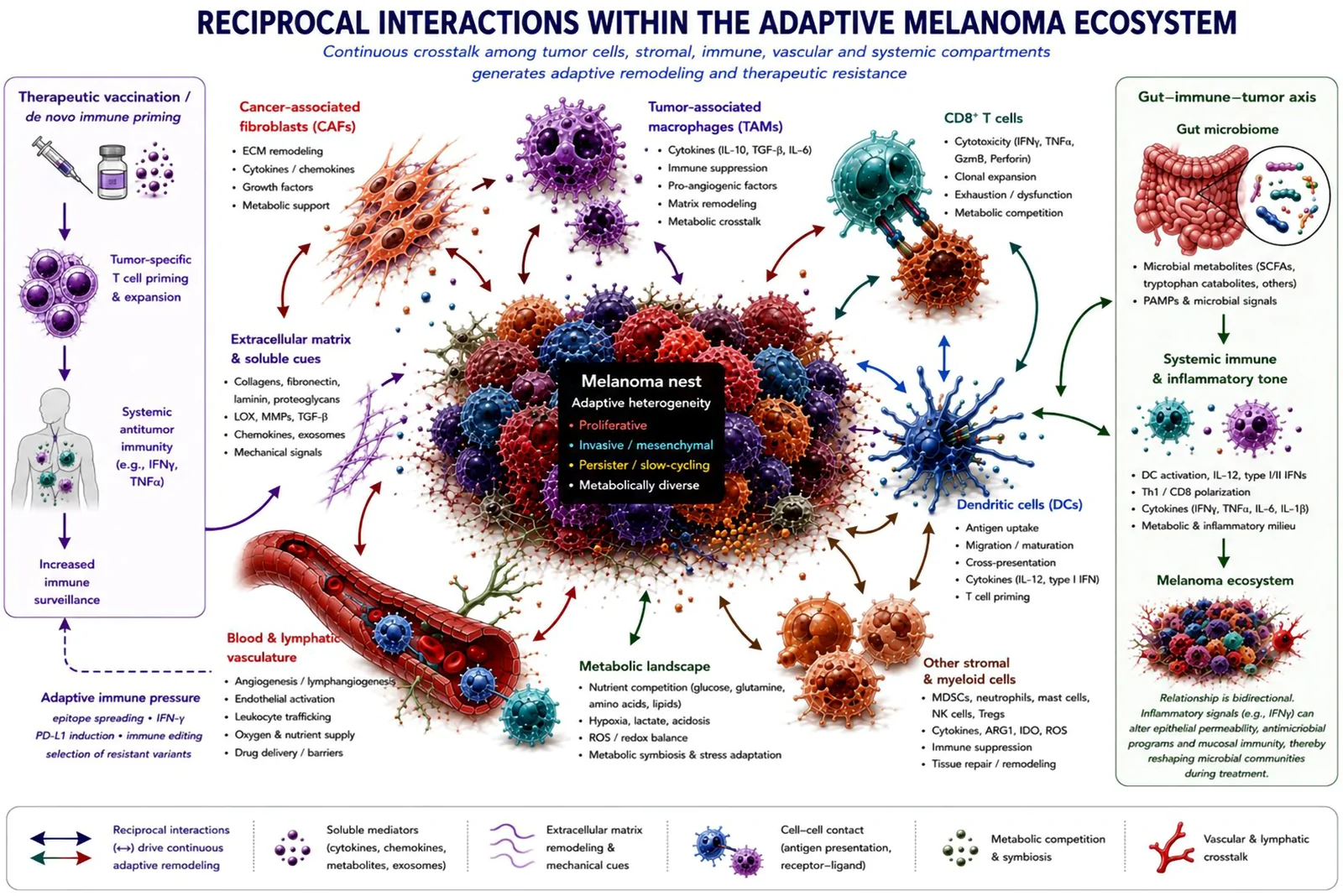
Reciprocal interactions within the adaptive melanoma ecosystem. Schematic representation of the reciprocal interactions linking heterogeneous melanoma cells with immune, stromal, vascular and metabolic components of the tumor microenvironment during therapeutic adaptation. Therapeutic vaccination and the gut microbiome illustrate complementary mechanisms through which anti-tumor immunity and systemic inflammatory signals may reshape this ecosystem. Symbols summarize the principal modes of intercellular and microenvironmental communication represented in the figure. Bidirectional connectors indicate established reciprocal functional interactions between the represented cellular or microenvironmental compartments, whereas unidirectional connectors indicate the predominant or specifically represented direction of functional influence in the context of the figure. Connector directionality does not imply a direct molecular interaction and does not exclude additional reciprocal effects not represented in the scheme. Connector colors are used primarily for visual organization.

One of the defining features of this reciprocal adaptation is competition for metabolic resources. Activated CD8^+^ T lymphocytes and natural killer (NK) cells require sustained glycolysis and mitochondrial metabolism to support proliferation, cytokine production and cytotoxic function. However, rapidly proliferating melanoma cells consume large amounts of glucose and amino acids, depriving immune cells of the nutrients necessary for effective anti-tumor responses (59, 80, 89, 93). Reduced glucose availability suppresses glycolytic activity and IFN-γ production by effector T cells, thereby limiting the efficacy of ICI (93, 94). Simultaneously, lactate accumulation, extracellular acidification, altered amino acid metabolism—including activation of the IDO–kynurenine pathway—and dendritic cell metabolic reprogramming promote regulatory T-cell expansion, tolerogenic myeloid phenotypes and impaired antigen presentation (83, 89, 94–97). Stromal populations, including cancer-associated fibroblasts, tumor-associated macrophages and endothelial cells, further reinforce this immunosuppressive niche through inflammatory cytokines, extracellular matrix remodeling and metabolic support (98–103).

Metabolic heterogeneity further amplifies this complexity. Distinct metabolic niches coexist within individual melanomas, generating localized regions of hypoxia, nutrient deprivation and oxidative stress that differentially shape immune-cell function and therapeutic responsiveness (82). Thus, metabolic adaptation should not be viewed simply as a mechanism sustaining tumor-cell survival, but as a process that simultaneously remodels the immune landscape and determines the quality of anti-tumor immunity. This reciprocal immunometabolic network provides the biological framework for the IFN-γ–NAMPT axis discussed below, in which inflammatory signaling and NAD^+^ metabolism become mechanistically integrated.

### 3.6 The IFN-γ–NAMPT axis links inflammation, metabolism and immune escape

Metabolic adaptation also determines how melanoma cells respond to inflammatory cytokines. Although IFN-γ is classically considered a key mediator of anti-tumor immunity, prolonged or dysregulated IFN-γ signaling may paradoxically promote adaptive resistance by inducing immune checkpoint molecules, including PD-L1 and PD-L2, thereby facilitating immune evasion (5, 37, 104). Consequently, the biological outcome of IFN-γ signaling is highly context-dependent and reflects the balance between acute immune activation and chronic inflammatory stimulation within the TME. We recently demonstrated that IFN-γ induces NAMPT through a Bromodomain and Extra-Terminal motif epigenetic factor (BRD4)-IRF1-dependent transcriptional program; in turn, NAMPT-dependent NAD metabolism supports mitochondrial complex I activity, STAT1 signaling and BRD4 recruitment to IFN-responsive promoters, including CD274/PD-L1 and NAMPT itself. This feed-forward circuit links cytokine signaling, chromatin regulation and mitochondrial metabolism (105).

Functional co-culture experiments showed that manipulating NAMPT in melanoma cells alters T-cell migration and cytotoxicity, demonstrating that tumor NAD metabolism actively shapes anti-tumor immunity. NAMPT expression also correlates with IFN-γ-responsive signatures and PD-L1 in patient cohorts. The net therapeutic consequence is context dependent: NAMPT can reinforce adaptive immune resistance, yet high IFN-γ/NAMPT states may also identify inflamed tumors with greater susceptibility to checkpoint blockade. These findings caution against assuming that metabolic inhibition will have identical effects across tumor and immune compartments.

## 4 Therapeutic vaccination as a strategy to reshape the adaptive landscape

Therapeutic vaccination occupies a distinctive position among strategies aimed at closing the adaptive window. While conventional therapies primarily inhibit an oncogenic pathway or release an inhibitory checkpoint, vaccines aim to actively stimulate patient’s own immune system to generate or expand tumor-specific and long-term immune responses. These responses can promote direct recognition and elimination of malignant cells but also initiate a broader remodeling of the TME, influencing antigen presentation, cytokine production, immune-cell recruitment, and the balance between immune activation and immune suppression (106–108), as depicted in **Figure 3**. Therefore, therapeutic vaccines have the potential to convert immunologically “cold” tumors into more inflamed lesions, eventually making them more treatment susceptible. Nevertheless, their limited efficacy as monotherapy illustrates that immune priming alone is rarely sufficient to overcome the multiple mechanisms of immune escape that characterize established melanoma.

### 4.1 Therapeutic vaccination: from immune activation to rational combination strategies

Melanoma has long served as a model for cancer vaccination because of its mutational burden and abundance of well-characterized antigens (109, 110). Over the past three decades, numerous therapeutic vaccines have been developed against both shared melanoma-associated antigens and patient-specific neoantigens. Among the most extensively investigated shared antigens are gp100, MART-1 (Melan-A), tyrosinase and tyrosinase-related proteins (TRP1/TYRP1 and TRP2/DCT), MAGE family antigens, NY-ESO-1, survivin, and, more recently, CSPG4 (111–114). These platforms employed different vaccine technologies, including peptides, dendritic cells, DNA plasmids, viral vectors, and mRNA formulations, and frequently elicited measurable antigen-specific immune responses. However, their clinical activity as monotherapies could be further improved. The main limitation is not necessarily a failure to generate reactive lymphocytes, but their inability to infiltrate tumors, maintain function and withstand antigen loss, checkpoint induction, metabolic competition and suppression by myeloid or stromal populations.

Vaccination can itself create adaptive immune pressure. Newly primed T cells exert IFN-γ production and tumor recognition, but also select antigen-negative cells and might induce PD-L1 and other inhibitory mechanisms. This feedback provides a strong rationale for combination rather than stand-alone vaccination.

### 4.2 Vaccination and checkpoint blockade: complementary immune functions

While ICIs predominantly reinvigorate pre-existing anti-tumor T cells and are less effective in immune-desert or immune-excluded tumors, anti-cancer vaccines address this limitation by inducing *de novo* priming or expansion of tumor-specific immune cells, promoting epitope spreading, and broadening the antigenic repertoire recognized by the immune system. In this division of labor, vaccines initiate and broaden immunity, whereas ICIs preserve effector function and limit adaptive checkpoint-mediated attenuation. This complementary biology provides the rationale for integrating vaccination with ICI to achieve more durable disease control and delay the emergence of therapeutic resistance.

Preclinical studies in melanoma models show that PD-1/PD-L1 blockade increases the expansion, persistence and intratumoral activity of vaccine-induced T cells, enhancing tumor control (115–118). These preclinical observations offered the biological foundation for subsequent clinical development of vaccine–ICI combinations which has now provided promising proof of concept. In the phase IIb KEYNOTE-942 study, the individualized neoantigen mRNA vaccine mRNA-4157/V940 combined with pembrolizumab improved recurrence-free survival in resected high-risk melanoma (119–121). In parallel, early studies of shared-antigen platforms such as BNT111 in checkpoint-refractory disease further support the principle that vaccination expands the substrate upon which checkpoint blockade can act, as evidenced by the NCT04526899 clinical trial.

### 4.3 Vaccination during the immunological window created by MAPK inhibition

BRAF/MEK inhibition rapidly but transiently reshapes the TME. Early treatment can increase melanoma differentiation-antigen expression, MHC-mediated presentation, dendritic-cell activity and CD8^+^ infiltration, while decreasing selected immunosuppressive cytokines and myeloid populations (122–125). This early phase of MAPK inhibition therefore represents a biologically defined window in which therapeutic vaccination may achieve maximal efficacy by exploiting improved antigen visibility and a more permissive immune context. This has broadened the spectrum of rational combinatorial strategies, positioning therapeutic vaccination as a promising partner not only for immunotherapy but also for targeted treatments in melanoma. Nevertheless, this favorable configuration is not durable. Progression can be accompanied by reduced antigen expression, diminished CD8^+^ T-cell infiltration and induction of immune checkpoints or dedifferentiated states. MEK inhibition may also impair priming of naïve lymphocytes while preserving the activity of pre-activated tumor-infiltrating cells, underscoring the importance of cellular compartment and treatment timing (126–128). The next challenge is the identification of the relevant molecular targets that actively participate in the adaptive response to BRAF/MEK inhibition. Vaccines directed against such resistance-associated antigens could exploit the transient immune-permissive state induced by MAPK inhibition while simultaneously targeting cellular programs responsible for disease persistence and relapse. Among these tumor-associated antigens, CSPG4 is an attractive candidate because of its broad expression in melanoma, limited distribution in normal adult tissues, and functional contribution to proliferation, migration, invasion and survival signaling (114, 129–132). Emerging evidence further suggests that CSPG4 expression is dynamically regulated during adaptation to MAPK inhibition, supporting the concept that targeted therapy may transiently enhance the therapeutic vulnerability of CSPG4-positive melanoma cells (26, 133–135). Unlike patient-specific neoantigens, CSPG4 also offers the practical advantages of a shared antigen while remaining functionally linked to tumor fitness, thereby potentially reducing the likelihood of biologically neutral antigen-loss variants. Collectively, these observations provide a biological rationale for combining BRAF/MEK inhibitors with CSPG4-directed vaccination, whereby targeted therapy may establish a permissive immunological landscape that can be consolidated by durable antigen-specific immune responses. Although direct evidence for optimal sequencing remains limited, this model provides a compelling framework for schedule-dependent combinations of targeted therapy and therapeutic vaccination.

## 5 The gut microbiome as a systemic regulator of melanoma adaptation

The gut microbiome extends the adaptive landscape beyond the tumor itself. Microbial communities and metabolites influence dendritic-cell maturation, antigen presentation, T-cell priming, cytokine production and epithelial barrier integrity, thereby establishing a systemic immune and metabolic context particularly relevant for ICI response. The microbiome is not a melanoma resistance mechanism in a narrow sense, but a host-level determinant of the pressure imposed by therapy and of the immune resources available to sustain tumor control (**Figure 3**).

### 5.1 Evidence linking gut microbiota to ICI response

Seminal mouse studies established causality. In a seminal paper published in Science in 2015, Sivan and colleagues observed that genetically identical C57BL/6 mice obtained from different vendors displayed markedly different rates of B16 melanoma cell growth and response to PD-L1 blockade, which correlated with distinct gut microbial communities. These differences were abolished following cohousing or fecal microbiota transfer, demonstrating that the phenotype was microbiota-dependent rather than genetically determined (136). Vétizou et al. also demonstrated that CTLA-4 blockade required an intact microbiota and that selected *Bacteroides* species restored therapeutic efficacy through dendritic-cell, Interleukin (IL)-12 and Th1 responses (137).

Clinical cohorts subsequently associated ICI response with microbiota composition. Gopalakrishnan et al. reported greater diversity and enrichment of *Ruminococcaceae/Faecalibacterium* in anti-PD-1 responders, accompanied by increased antigen presentation and CD8^+^ T-cell infiltration (138). Matson et al. identified a partially distinct responder-associated signature including *Bifidobacterium longum, Collinsella aerofaciens and Enterococcus faecium* (139). Although the identified taxa differed between studies, both papers demonstrated that favorable microbial ecosystems promote enhanced antigen presentation, greater CD8^+^ T-cell activation and improved clinical outcomes in melanoma patients.

### 5.2 Microbial control of IFN-γ-dependent immunity and metabolism

The microbiome can act upstream of IFN-γ by promoting pattern recognition receptors (PRR)-dependent dendritic-cell activation, IL-12 production and Th1/CD8^+^ polarization (140). Microbiota from melanoma patients responding to PD-1 blockade increases IFN-γ-producing lymphocytes and improves checkpoint blockade when transferred into germ-free mice (136). Microbial metabolites provide additional signals: inosine can support Th1 differentiation under appropriate costimulation (141), while short-chain fatty acids exert context-dependent effects on regulatory and memory T cells (142–145).

This relationship may be bidirectional. IFN-γ alters epithelial permeability, antimicrobial programs and mucosal immune composition, potentially reshaping microbial communities during treatment (146–148). Longitudinal studies in advanced melanoma patients have shown that gut microbial communities undergo significant remodeling during ICI therapy and suggest that treatment-associated microbial trajectories may predict durable benefit better than baseline composition alone (149).

Based on these findings, it seems plausible that the gut microbiome, modulating the intensity and duration of IFN-γ signaling, may indirectly contribute to melanoma metabolic adaptation including changes in NAMPT/NAD metabolism and mitochondrial redox activity.

### 5.3 Therapeutic microbiome modulation

Fecal microbiota transplantation (FMT) currently provides the strongest clinical proof of concept. In anti-PD-1-refractory melanoma, FMT from responding donors followed by pembrolizumab rechallenge produced clinical benefit in a subset of patients and was associated with donor engraftment, increased CD8^+^ T-cell activation and reduced immunosuppressive myeloid populations (150, 151). FMT from healthy donors has also shown feasibility and encouraging activity in treatment-naïve patients (152).

Defined bacterial consortia, live biotherapeutics, dietary fiber and prebiotic interventions may offer more standardized alternatives, but randomized evidence remains limited (153–157). Microbiome modulation should therefore be viewed as a promising complementary strategy rather than established clinical practice. Its eventual use will require reproducible functional biomarkers, standardized manufacturing and careful consideration of antibiotics, diet, geography and host genetics.

## 6 Computational integration of the adaptive trajectory

Artificial intelligence (AI) and machine learning are now applied across the melanoma pathway, from lesion detection to prediction of therapeutic response. Their relevance to the framework proposed here lies in a specific capability rather than in diagnostic performance as such. That capability is the integration of heterogeneous, serially acquired measurements into a single estimate of tumor state. The readouts required to locate a tumor within the adaptive trajectory include serial histology, imaging, single-cell and spatial profiles, and metabolic and immune parameters. These readouts are individually uninformative and jointly difficult to interpret manually.

In melanoma the most consolidated applications remain image-based: convolutional networks, and more recently vision transformers and multimodal architectures. These models now classify pigmented lesions with accuracy comparable to that of experienced dermatologists. The largest and most reproducible gains are obtained when algorithmic output is combined with clinical judgment rather than substituted for it (158, 159). Analogous approaches have moved from pixels to tissue. Computational pathology can segment whole-slide images into tumor, epidermal and TME compartments. It can also quantify features such as mitotic activity, which are excluded from staging systems because of inter-observer variability (160). Such tools provide a reproducible substrate on which the immune and stromal changes discussed above may be measured.

At the molecular level, attention-based models applied to bulk transcriptomes and multi-omics classifiers integrating genomic and transcriptomic features predict response to checkpoint blockade more accurately than mutational burden alone. Through interpretability methods, these models also nominate the pathways underlying their own predictions (161, 162). Longitudinal applications are less mature, but conceptually closer to our argument. Models trained on serial imaging or repeated molecular sampling aim to identify the transition from a stable to an evolving state before it becomes clinically apparent (163). Reinforcement-learning approaches have been used, so far only in silico, to derive dosing schedules that maintain tumor control with minimal cumulative exposure (164). This is precisely the class of question raised by intermittent and sequential regimens.

These applications remain provisional. Most supporting evidence is retrospective and single center. Training sets under-represent darker phototypes and rare subtypes, and molecular cohorts are small. The opacity of the best-performing models also complicates their use for decisions about timing rather than diagnosis. Recent regulatory frameworks classifying medical AI as high-risk, and requiring transparency and post-market monitoring, reflect these concerns (165). Applied to the adaptive window, machine learning is therefore best regarded not as a source of new biology, but as a way of reading known biology at adequate temporal resolution. Its value will depend on prospective validation in treated patients rather than on further gains in benchmark performance.

## 7 Closing the adaptive window: principles for rational combination and therapeutic scheduling

The evidence discussed throughout this Review suggests that therapeutic adaptation is not a single molecular event but a temporally evolving physiological process. Rather than arising abruptly through genetically fixed resistance mechanisms, melanoma initially responds to therapeutic pressure through coordinated remodeling of signaling, metabolism, inflammatory responses, cell-state plasticity and interactions with the TME. These adaptive programs preserve sufficient cellular fitness to allow the subsequent emergence and selection of more stable resistant states.

The adaptive window hypothesis can be tested by asking whether therapeutic perturbations leave multiple viable adaptive trajectories accessible and whether candidate interventions reduce that accessibility. Current evidence supports co-existing or distinct routes to drug tolerance and shows that priming, cell-state transitions and trajectory positioning can be experimentally modified, but it does not establish a universal molecular machinery that maintains adaptive capacity. Whether the accessibility of adaptive trajectories depends on shared permissive regulators, on context-specific and partially redundant networks, or emerges from distributed interactions among tumor and microenvironmental processes therefore remains unresolved. Perturbational approaches should distinguish interventions that suppress one realized adaptive phenotype from those that reduce the range of viable responses generated under therapeutic pressure. The latter outcome would provide direct support for the adaptive window hypothesis and an operational criterion for window closure.

To operationalize these distinctions longitudinally, **Table 1** relates observable tumor conditions and candidate biological features to their interpretation in terms of adaptive capacity and potential therapeutic implications.

**Table 1:** Longitudinal biological and operational framework of the adaptive window (AW) in melanoma.

Observable condition	Biological characteristics	Candidate longitudinal features	Adaptive-capacity interpretation	Therapeutic implication	AW therapeutic opportunity	Stabilization of resistance
Pre-treatment state	Pre-treatment oncogenic, metabolic, immune and cell-state configuration.	Tumor burden; pathway activity; antigen expression; immune infiltration; baseline cell-state and metabolic features.	Baseline phenotype alone does not establish whether adaptive capacity is high or low. It provides the reference against which treatment-induced changes are interpreted.	Establish reference state and initiate the primary therapy according to disease context.		
Early adaptive remodeling	Rapid and potentially reversible signaling, metabolic, inflammatory and phenotypic remodeling induced or reinforced by therapy.	RTK/PI3K-AKT or FAK rebound; TBK1-interferon-associated programs; NAMPT-dependent metabolic and redox shifts; PD-L1 and antigen changes; transitions among MITF-high, AXL-high, neural crest-like or slow-cycling states.	Evidence that adaptive processes are engaged. Alternative viable trajectories may remain accessible; no single observed response is assumed to define the full Adaptive Window.	Intercept validated enabling dependencies or treatment-induced vulnerabilities at the earliest biologically rational and safely targetable point.		
Drug-tolerant persistence	Survival of residual populations under treatment, often with slow cycling and heterogeneous phenotypic, signaling and metabolic programs.	Persister-associated cell states; MITF/AXL/SOX10-related programs; slow-cycling features; metabolic dependencies NAMPT, OXPHOS; residual signaling or inflammatory programs.	Persistence does not necessarily mark the end of the Adaptive Window. The window may remain biologically open if residual populations retain access to alternative viable trajectories.	Target validated persister dependencies and, where demonstrable, residual adaptive capacity rather than assuming a single fixed persister state.		
Progressive stabilization	Reduced reversibility; persistent transcriptional or epigenetic memory; clonal selection/expansion; acquisition or enrichment of genetic alterations that sustain resistance.	Stable pathway reactivation; persistent antigen loss or immune-evasive features; durable transcriptional/epigenetic states; expansion of resistant clones.	Adaptive flexibility is increasingly constrained as one or more resistant solutions become stabilized. The relative relevance of reversible adaptive capacity declines, although different lesions or subclones may remain asynchronous.	Resistance-directed strategies increasingly predominate; residual adaptive processes may still require context-specific assessment.		

The opposing color scales are schematic and do not imply a monotonic temporal change in AW therapeutic opportunity. Depending on the therapeutic context, adaptive capacity and associated therapeutic opportunities may emerge or increase following treatment-induced perturbation before declining as resistance becomes progressively stabilized.

This perspective changes the rationale for therapeutic intervention (**Figure 4**). The most effective therapeutic partner may not necessarily target the dominant pathway before treatment, but the biological dependencies created by the initial therapeutic perturbation. Accordingly, the appropriate timing of a therapeutic partner should depend on the biology of the adaptive dependency being targeted. Upfront or concomitant treatment may be appropriate when a permissive dependency is already operative, whereas sequential intervention may be preferable when a targetable vulnerability is induced by the initial therapy. Vaccination, microbiome modulation and metabolic intervention further expand this framework by acting on complementary components of the adaptive response rather than on isolated resistance mechanisms.

**Figure 4:**
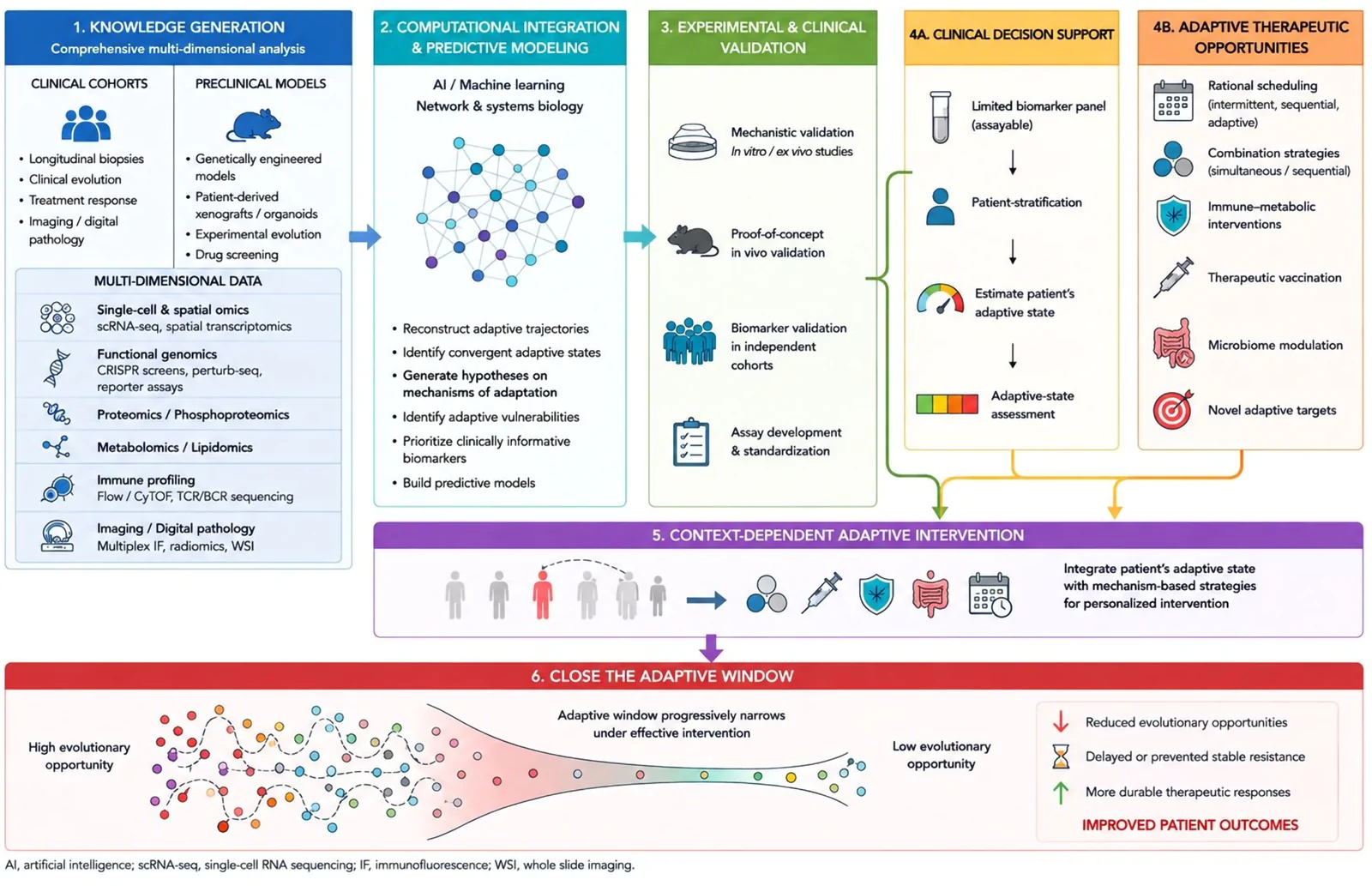
Closing the adaptive window: from knowledge generation to adaptive intervention. Conceptual framework for translating longitudinal biological information into context-dependent strategies aimed at constraining melanoma adaptation. Multidimensional data from preclinical models and clinical cohorts provide a discovery space in which AI/ML-assisted integration may support feature selection and the identification of candidate biomarkers of adaptive capacity. Following independent biological and clinical validation, reduced biomarker sets could support longitudinal monitoring and inform the rational timing and selection of therapeutic interventions. The framework is conceptual and does not imply that AI/ML independently defines adaptive biology or establishes therapeutic actionability.

Importantly, biologically informed timing should not be equated with fixed intermittent treatment. Randomized studies of intermittent MAPK inhibition have not demonstrated a clinical advantage over continuous therapy. In S1320 (https://clinicaltrials.gov/study/NCT02196181), continuous dabrafenib plus trametinib produced numerically longer post-randomization progression-free survival than a prespecified intermittent schedule, while intermittent vemurafenib plus cobimetinib failed to improve progression-free survival in https://clinicaltrials.gov/study/NCT02583516. These findings argue against fixed, unselected treatment withdrawal as a general strategy, while leaving open the distinct possibility that therapeutic vulnerabilities could be exploited when intervention is linked to a biologically defined adaptive dependency rather than to a predetermined calendar schedule.

A major challenge, however, is translating this conceptual framework into clinical practice. Comprehensive longitudinal characterization using serial biopsies, multi-omics, spatial analyses and functional imaging is unlikely to be feasible for routine management of every patient. Instead, these approaches should be viewed primarily as discovery platforms capable of defining the adaptive landscape and identifying the biological variables that most faithfully report tumor evolution.

Several practical limitations must be addressed before the adaptive window framework can inform clinical decision-making. The onset, duration and biological boundaries of the adaptive phase are unlikely to be uniform across patients, metastatic sites or treatment regimens, and adaptive programs may emerge asynchronously across tumor and microenvironmental compartments. Consequently, a single time point may capture a dominant adaptive state without establishing whether alternative viable trajectories remain accessible. Moreover, no real-time biomarker has yet been validated to track adaptive capacity or determine when a relevant adaptive dependency is biologically operative and reversible. Importantly, suppression of an individual adaptive response would not by itself demonstrate restriction of the adaptive window, which would require evidence that adaptive capacity or the accessibility of viable adaptive trajectories has been reduced. Any therapeutic strategy derived from this framework would subsequently require conventional evaluation of its pharmacological and clinical feasibility. Modality-specific constraints discussed above—including pleiotropic effects across tumor and immune compartments and the reproducibility and standardization challenges of microbiome-directed approaches—would also need to be considered.

Clinical translation of the adaptive window framework will require a stepwise validation strategy (**Figure 5**). Preclinical studies should first establish whether adaptive capacity can be operationally measured and experimentally restricted, rather than merely documenting suppression of a dominant adaptive response. Molecular features associated with experimentally defined adaptive capacity could then support artificial intelligence/machine learning (AI/ML)-assisted discovery of candidate multivariate signatures in longitudinal bulk or single-cell datasets, followed by independent biological and clinical validation. If validated, such signatures could serve both to monitor the biologically active adaptive window and as pharmacodynamic readouts of interventions designed to constrain it. Importantly, therapeutic benefit alone would not demonstrate window closure, because improved outcome could result from inhibition of a single dominant resistance pathway; evidence that adaptive capacity itself has been reduced would provide a complementary mechanistic test of the framework.

**Figure 5:**
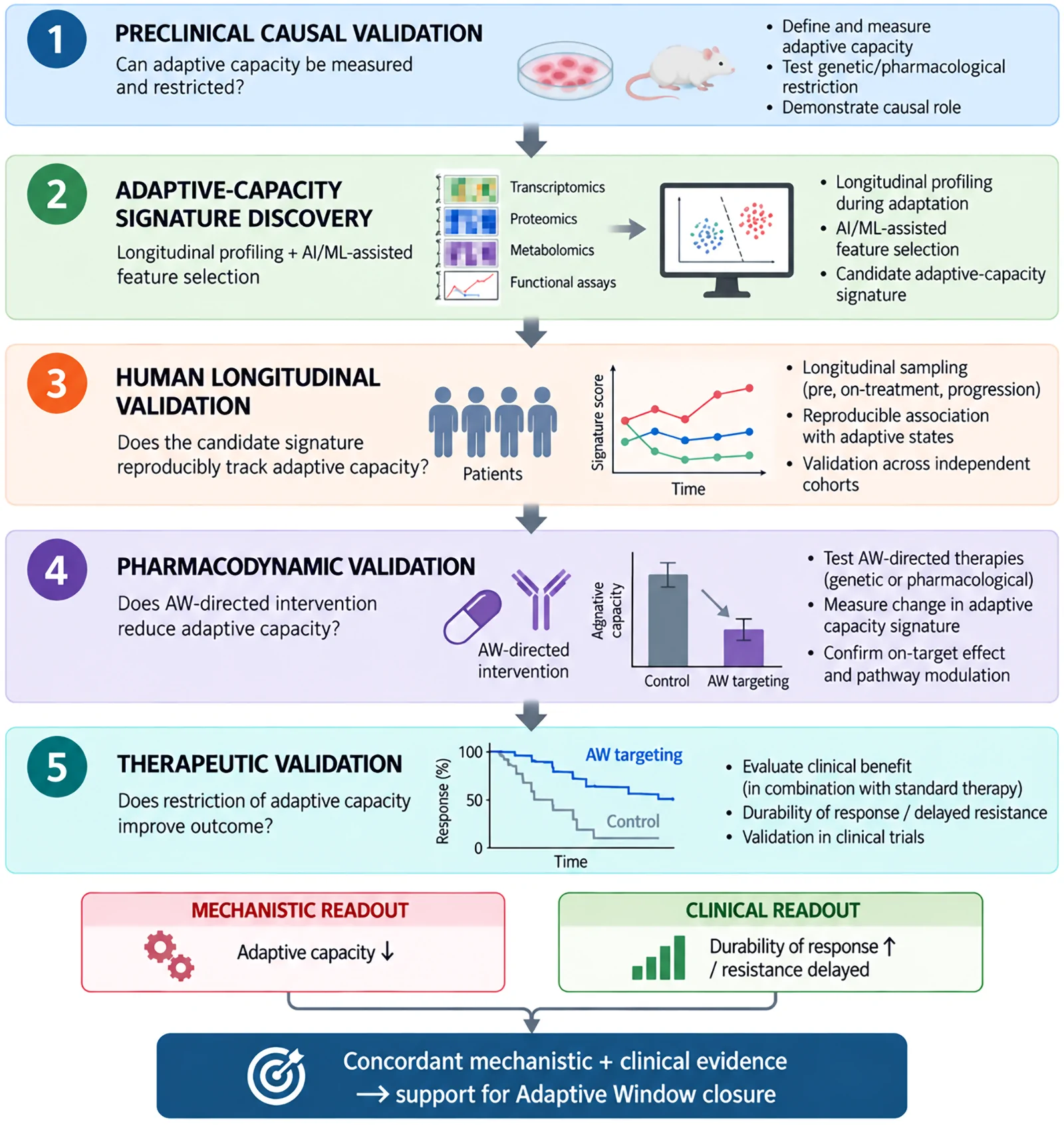
Validation framework for adaptive window (AW) closure. A stepwise preclinical-to-clinical framework to define, identify, and validate adaptive capacity, from causal preclinical studies and signature discovery to longitudinal human validation, pharmacodynamic assessment, and therapeutic validation. Concordant mechanistic evidence of reduced adaptive capacity and clinical evidence of increased response durability or delayed resistance provide support for adaptive window closure.

The adaptive window should therefore be understood as a biologically defined adaptive phase rather than as a predetermined temporal interval after treatment initiation. Because response and resistance kinetics vary across patients, lesions and therapeutic contexts, an adaptive window-directed intervention should be introduced at the earliest point at which the relevant adaptive dependency is biologically operative and safely targetable. This may favor upfront or concomitant treatment when a shared enabling dependency is already present, or biology- and biomarker-informed sequential treatment when a targetable dependency is induced by the initiating therapy. Within this validation framework, AI/ML has a specific role in candidate biomarker discovery rather than in defining the underlying biology itself. Molecular profiles obtained from systems in which adaptive capacity has been experimentally characterized could provide the discovery space for multivariate signatures, while AI/ML-assisted integration of longitudinal bulk or single-cell transcriptomic data and complementary measurements may facilitate feature selection and dimensionality reduction. Any resulting signature should be regarded as a candidate biomarker until independently validated biologically and clinically. If validated, a reduced signature could subsequently support longitudinal monitoring and pharmacodynamic assessment without requiring exhaustive molecular profiling in routine practice (**Figure 5**).

### 7.1 Conclusions

The central message of this Review is that therapeutic resistance should not be viewed exclusively as the consequence of heterogeneous genetic alterations, but as the outcome of a preceding adaptive phase during which melanoma cells and their surrounding ecosystem retain the capacity to compensate for therapeutic stress. The adaptive window hypothesis proposes that resistance is facilitated not only by individual adaptive responses, but by the continued capacity of the tumor to access alternative viable adaptive trajectories.

This hypothesis predicts that effectively closing the adaptive window should restrict the diversity or accessibility of viable adaptive trajectories, rather than merely suppressing the response that is dominant at a given time. Whether this can be achieved by targeting shared dependencies that enable adaptation or will require context-specific combinations remains to be established. Rather than targeting the heterogeneous endpoints of tumor evolution, future therapeutic strategies may achieve greater clinical benefit by constraining the convergent adaptive processes that precede stable resistance.

Should the adaptive window hypothesis be experimentally validated in melanoma, an important question will be whether the same principle extends to other malignancies in which therapeutic pressure elicits substantial adaptive plasticity before stable resistance. If so, constraining the capacity to generate viable adaptive trajectories, rather than targeting their heterogeneous resistant endpoints, could represent a more general principle for limiting therapeutic escape.
